# Multifunctional Modified Tumor Cell Membranes-Coated Adjuvant PTX against Melanoma

**DOI:** 10.3390/biom13010179

**Published:** 2023-01-14

**Authors:** Zhonghua Ji, Bingying Lin, Enshuang Guan, Mingsen Zhou, Hui Wang, Ying Hu

**Affiliations:** 1 Pharmacy, Zhejiang Pharmaceutical College, Ningbo 315000, China; 2 School of Pharmaceutical Sciences, Wenzhou Medical University, Wenzhou 325000, China

**Keywords:** cell membrane, PTX, fusion peptide

## Abstract

Melanoma is the deadliest type of skin cancer. Anti-tumor immunotherapy has made great progress in increasing the overall survival of patients. However, many physiological barriers cause low bioavailability of drugs. Cell membranes are becoming increasingly prevalent for assisting drug delivery because of the significant benefits of avoiding host cell barriers. Herein, B16F10 cell membranes (BFMs) were prepared in this study. BFMs could not only act as antigens but also serve as vesicles for vaccines. To trigger potent immunity, BFMs must be taken up by dendritic cells (DCs) and combined with adjuvants to make BFMs overcome the immune tolerance. To avoid circulating BFMs into tumors and quickly internalized by DCs after subcutaneously injection, the antigen-cell penetrating fusion peptide WT(YGRKKRRQRSRRYVDFFVWL) was used to modify BFMs. Additionally, a low dosage of paclitaxel (PTX) can activate DCs via toll-like receptor-4 (TLR-4). Therefore, we developed PTX-loaded micelles using Pluronic^®^ F127. Then, WT-modified BFMs (WT-BFMs) were coated F127-PTX to yield WT-BFMs/ F127-PTX. Optimized WT-BFMs/F127-PTX promoted the cellular uptake and showed remarkable efficacy in eliciting robust antigen-specific cellular and humoral immune responses.

## 1. Introduction

Melanoma is the deadliest skin cancer, and its morbidity and mortality rates continue to rise [1]. Anti-tumor immunotherapy has made great progress in increasing the overall survival of patients. Tumor-specific peptide, protein, RNA and DNA are typical vaccine antigens. However, for different patients, these vaccines show significantly varied immunizations owing to the highly specific antigens themselves [2]. Additionally, many physiological barriers cause low bioavailability and poor targeting of drugs [3]. Thus, to overcome the limitations of typical vaccine antigens, whole tumor cell lysates or cell membranes with mixed tumor-associated antigens have been chosen [4].

As a new drug delivery system, cell membranes are known to circumvent the limitations of conventional nanoparticles [5,6]. However, in some studies, tumor cell-derived membranes have been harnessed due to unexpected immune suppression [7]. Because of homotypic adhesion properties, the targeted mother-cells characteristic of tumor cell membranes, their surface proteins were not enough to induce recognition by dendritic cells(DCs) which are the most effective antigen presenting cells (APCs). Developing effective immunotherapies with high targeting and tumor specificity is the goal of melanoma treatment [8]. To trigger potent immunity, adjuvants are required for cancer vaccines to overcome the immune tolerance [9,10]. Significant improvement is warranted. Therefore, we explored the use of functional murine B16F10 cell-derived membranes (BFMs) to deliver adjuvants and achieve fast uptake by DCs.

Cell-penetrating peptide (CPP) facilitate the internalization of different cargo into certain cells. TAT (YGRKKRRQRRR) is a classical canonic CPP [11]. Trp2 (SVYDFFVWL) is a classical hydrophobic melanoma-specific antigen [12,13]. Herein, Trp2 (SVYDFFVWL) was combined with hydrophilic TAT (SVYDFFVWL) by a covalent reaction to yield the functional fusion peptide WT (YGRKKRRQRSRRYVDFFVWL) [12]. The synthetic method is a conventional method for modifying particles with TAT, which may affect the biological nature of biomimetic nanoparticles. The obtained WT is an amphiphilic peptide that can be inserted into BFMs without synthesis or the use of organic reagents.

To be a successful vaccine delivery system, a robust adjuvant for triggering potent cellular immune responses is needed [14]. Paclitaxel (PTX) is an effective chemical drug against melanoma that stabilizes microtubules to prevent cell division and cause cell death. At sub-therapeutic doses, PTX could mimic toll-like receptor (TLR) agonist LPS to promote the maturation of DCs via TLR-4, leading to the secretion of pro-inflammatory cytokines such as IL-6, IL-12, and TNF-α and increased expression of major histocompatibility complex class II (MHC-II) [15]. Therefore, PTX is an excellent candidate for adjuvant therapy. Pluronic^®^ F127 is a triblock polymer that can form micelles [16]. In this study, hydrophobic PTX was encapsulated into F127 micelles for delivery. 

**Figure 1:**
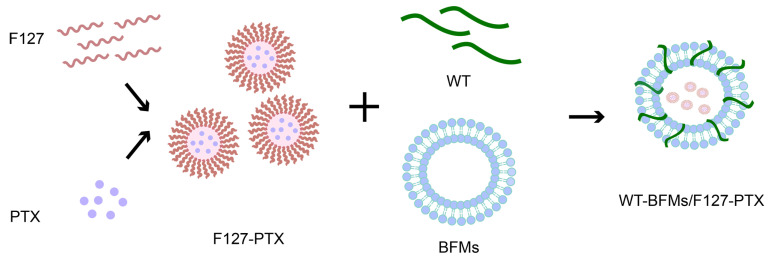
Schematic illustration of WT-BFMs/F127-PTX. PTX-loaded F127 micelles were prepared by the thin-film dispersion method. WT, F127-PTX micelles and BFMs were mixed and sonicated using ultrasonic homogenizer to form WT-BFMs/F127-PTX.

Owing to the unique immunological characteristics of the skin, many Langerhans cells are located in the epidermis of the outermost layer of the skin. The goal of the present trial was to design and characterize a novel multi-drug delivery system and evaluate whether this system could generate robust anti-tumor immunity upon subcutaneous immunization. Based on the above studies, we utilized an emerging nanotechnology based on cell membranes (Figure 1). We fabricated PTX-loaded and fusion peptide WT-modified BFMs (WT-BFMs/F127-PTX). This modified tumor-cellular membrane delivery approach is multifaceted, offering antigen characteristics, improved internalization capability, and simultaneous multi-drug delivery. In vitro cell assays were performed to validate this concept. Here we subcutaneously injected WT-BFMs/F127-PTX or control groups and carried out in vivo immunity assays of CD4^+^ T and CD8^+^ T cells secreting cytokines, as well as other assays.

## 2. Materials and Methods

### 2.1. Materials

WT (YGRKKRRQRSRRYVDFFVWL, Purity ≥ 95%) and FITC-WT (FITC-YGRKKRRQRSRRYVDFFVWL, purity ≥ 95%) were synthesized by GL Biochem, Ltd. (Shanghai, China). Pluronic^®^ F127 (molecular weight 12,600 Da), and 3-(4,5-dimethylthiazol-2-yl)-2,5-diphenyltetrazolium bromide (MTT) were purchased from Sigma-Aldrich (St. Louis, MO, USA). DAPI staining solution, phenylmethanesulfonylfluoride(PMSF), enhanced BCA protein assay kit and membrane and cytosol protein extraction kit were obtained from Beyotime Biotechnology (Shanghai, China). Recombinant murine Mouse GM-CSF was obtained from Absin (Shanghai, China). FITC labeled CD11 c+, PE-labeled CD86, PE-labeled MHC class II, PD-1 and PE-CY5 labeled FOXP3 FJK-16S monoclonal antibodies were obtained from Invitrogen (Carlsbad, CA, USA). IFN-γ, interleukin-4 (IL-4), and interleukin-12 p70 (IL-12 p70), mouse uncoated enzyme-linked immunosorbent assay (ELISA) kits with plates were obtained from Invitrogen (Carlsbad, CA, USA). The FITC-labeled CD40 monoclonal antibody was purchased from BD Pharmingen (Franklin Lakes, NJ, USA).

### 2.2. Cell Lines & Cell Culture

A murine melanoma cell line B16F10 cells were maintained in RPMI-1640 medium (Gibco, Thermo Fisher Scientific, Grand Island, NY, USA) supplemented with 10% fetal bovine serum, penicillin and streptomycin (Invitrogen). DC 2.4 cells were also cultured in a RPMI-1640 medium supplemented with 10% fetal bovine serum and antibiotics.

Mouse bone marrow-derived DCs (BMDCs) from C57BL/6 mice were harvested as follows. Sterile femurs and tibiae were dissected. The marrow cells were harvested and red blood cells were lysed using ACK lysis buffer (0.15 M NH_4_Cl, 10.0 mM KHCO_3_, 0.1 mM EDTA, pH 7.4) for 10 min. The same volume of phosphate-buffered saline(PBS) was added to stop cell lysis. The obtained cells were cultured in a RPMI-1640 culture medium containing 10% FBS and antibiotics supplemented with recombinant murine mouse GM-CSF (20 ng/mL) and β-mercaptoethanol (50 μM) at 37 °C under a humidified atmosphere containing 5% CO_2_ on day zero. On day two, the nonadherent cells were washed, and fresh 10% FBS RPMI-1640 culture medium with GM-CSF (20 ng/mL) and β-mercaptoethanol (50 μM) was added. On days four and six, half of the medium volume was replaced with complete medium. On day six, loosely adherent DCs were harvested as immature DCs (iDCs). iDCs were then stained with FITC-labeled CD11c^+^ monoclonal antibody and analyzed using a BD Accuri C6 Plus flow cytometey (BD, Franklin Lake, NJ, USA). IFN-γ, IL-4, and IL-12 p70 mouse uncoated ELISA kit with plates were obtained from Invitrogen (Carlsbad, CA, USA).

### 2.3. Animals

Female C57BL/6 mice (six- to eight-week-old) were purchased from Zhejiang Laboratory Animal Center (Hangzhou, China). Mice were housed in specific pathogen-free, light-cycled, and temperature- and humidity-controlled facilities. All procedures conducted with animals were approved by the institutional animal care committee and were based on set guidelines.

### 2.4. Method

#### 2.4.1. F127/PTX Micelles Preparation and Characterization

PTX-loaded F127 micelles were prepared by the thin-film dispersion method. Briefly, PTX in methanol was added to the F127 acetonitrile solution (W/W = 1:10). The mixed solution was rotary-evaporated under vacuum at 40 °C for 20 min to eliminate the organic phase and form a film, which was then hydrated with PBS at 40 °C. The particle size, PDI, and surface charge of F127/PTX were measured using a Zeta-sizer Nano ZS 90 (Malvern Instruments, Malvern, UK). The concentration of encapsulated PTX was determined using high performance liquid chromatography(HPLC) at 227 nm. 

#### 2.4.2. B16F10 Membranes Preparation and Characterization

B16F10 cells were collected and lysed in hypotonic 0.25 × PBS lysis buffer consisting of 1% PMSF at 4 °C for 15 min. The solution was centrifuged at 1000× *g* for 10 min to remove extra cell debris, and the supernatants were collected and further centrifuged at 11,000× *g* for 30 min at 4 °C. The membrane fragments were resuspended, washed with cold PBS, and centrifuged (11,000× *g*, for 30 min, 4 °C). The final B16F10 cell-membrane (BFMs) pellet was stored in distilled water for subsequent experiments. The whole BFM protein was quantified using enhanced BCA protein assay kit (Beyotime, Shanghai, China).

#### 2.4.3. The Membranes Derivation Preparation and Characterization 

Different proportions of the WT solution were added to the BFMs solution and sonicated for 6 min using an ultrasonic homogenizer (SCIENTZ-Ⅱ D, Ningbo, China). The particle size, PDI, and surface charge of the obtained WT-BFMs were measured using a Zeta-sizer Nano ZS 90 (Malvern Instruments, UK).

Fluorescence microscopy was also used to visualize WT-modified BFMs. The WT-BFMs were prepared using FITC-WT and DiD-stained BFMs. Colocalization of the BFMs (red channel) and FITC-WT (green channel) were imaged, and the yellow color represents colocalization of BFMs and FITC-WT. Furthermore, the colocalization of BFMs and WT upon intracellular uptake by BMDCs for 4 h (the final concentration of WT was 20 μg/mL) was observed using fluorescence microscopy. Cell nuclei were labeled with 4,6-diamidino-2-phenylindole (DAPI; blue channel) (Beyotime, Beijing, China). 

#### 2.4.4. The Preparation of WT-BFMs Coated with F127-PTX Nanoparticles

WT, F127-PTX micelles and BFMs (4:1:4, W/W/W)were mixed and sonicated using an ultrasonic homogenizer to form WT-BFMs/F127-PTX. The physical characteristics of WT-BFMs/F127-PTX were determined using a Zeta-sizer Nano ZS 90 (Malvern Instruments, UK) and transmission electron micrographs. 

#### 2.4.5. Colocalization of the FITC-WT and BFMs

BMDCs were seeded into 12-well plates. The WT-BFMs were prepared using FITC-WT(green) and DiD-stained BFMs (red). WT-BFMs at a final concentration of 20 μg/mL WT were added and cultured for 4 h. The cells were then washed with PBS and fixed with 4% paraformaldehyde for 10 min. After the cells were washed, the colocalization of BFMs and FITC-WT was observed by fluorescence microscope.

#### 2.4.6. Cell Viability Assay

Immature BMDCs were harvested as previously described. DCs were incubated with WT-BFMs or WT-BFMs/F127-PTX at different concentrations for 48 h in 96-well plates, and the 3-(4,5-dimethyl-2-thiazolyl)-2,5-diphenyl-2H-tetrazolium bromide (MTT) assay was conducted. Briefly, MTT solution (5 mg/mL) was added, and the cells were incubated. Formazan crystals were dissolved in dimethyl sulfoxide(DMSO). The optical density(OD) of each well at a wavelength of 490 nm was measured using an ELISA reader. Cell viability (%) was calculated using the following formula.

Viability (%) = ([Abs]_sample_ − [Abs]_blank_)/([Abs]_control_ − [Abs]_blank_)

#### 2.4.7. Cellular Uptake Assay

DC 2.4 cells were seeded into 12-well plates. The WT-BFMs were prepared using WT and DiD-stained BFMs (red). WT-BFMs at a final concentration of 20 μg/mL WT or DiD-BFMs were added and cultured for 4 h. The cells were then washed with PBS and fixed with 4% paraformaldehyde for 10 min. After washing three times with PBS, the cell nuclei were stained with DAPI for 5 min. After the cells were washed, the uptake of BFMs and WT-BFMs in DC 2.4 was observed using fluorescence microscope.

#### 2.4.8. Cellular Uptake Mechanism Assay

The cellular uptake mechanism of the WT-BFMs and BFMs were analyzed using intracellular uptake assays at different ratios of WT-BFMs and BFMs or at different temperatures (4 °C and 37 °C) or uptake times. B16F10 and DC 2.4 cells were seeded into two 24-well plates at a density of 1 × 10^5^ cells/well and cultured overnight. The composition of the WT-BFMs was optimized using in vitro uptake assay. DiD-stained BFMs (BM) were mixed and co-sonicated with different proportions of WT solution (the final concentrations of WT were 5, 20, 40, 60, 80 and 100 μg/mL) to prepare the WT-BFMs. One plate was allowed to stand at 4 °C, and the other was incubated at 37 °C. After 30 min, the BFMs fused with different concentrations of WT were added to each well of two plates and cultured for 30 min. The cells were then treated with 0.25% trypsin and EDTA and washed with PBS. The cells were then dissociated, washed, and resuspended. Uptake efficiency was analyzed using a C6 flow cytometer (BD, USA). 

The uptake of WT-BFMs was also observed using fluorescence microscopy. BMDCs or DC 2.4 were seeded into 24-well plates (1 × 10^5^ cells/well). DiD-labeled WT-BFMs were added and co-cultured separately with cells for 0.5, 1, 2, and 4 h. After the cells treated with WT-BFMs for 0.5, 1, 2, and 4 h, cells were washed and fixed with 4% paraformaldehyde. After washed thrice times with PBS, the cell nuclei were stained with DAPI for 7 min at room temperature. Cells were then washed thrice with PBS and imaged using fluorescence microscopy. Additionally, the uptake efficiencies of BFMs and WT-BFMs were measured using a flow cytometer (BD, USA). 

#### 2.4.9. BMDCs Maturation and ELISA Assays

Immature BMDCs were harvested as previously described. Cells were seeded into 24-well plates and separately co-cultured with WT-BFMs/F127-PTX, WT-BFMs, BFMs, F127-PTX, or PTX for two days. Cells were also pulsed with LPS as a control. An activation of BMDCs was determined using a flow cytometer (BD, USA) by measuring the expression of CD40 and CD86, which are mature indicators of BMDCs. In another parallel experiment, cells were stained with PE-labeled MHC class Ⅱ monoclonal antibodies to analyze MHC-Ⅱ expression.

Cytokines IL-4 and IL-12 p70 produced by BMDCs were measured using ELISA kits. BMDCs were seeded into 24-well plate and co-incubated with WT-BFMs/F127-PTX, WT-BFMs, BFMs/F127-PTX, WT, or lipopolysaccharides (LPS) with the same concentration of BFMs or PTX for 48 h. The culture supernatants of the BMDCs were harvested and diluted to an appropriate concentration. Cytokine levels were measured using ELISA (Invitrogen, Waltham, MA, USA), according to the manufacturer’s instructions. 

#### 2.4.10. Mouse immunization

Female C57BL/6 mice aged six to eight-weeks were administrated the following formulations: (1) WT-BFMs/F127-PTX, (2) WT-BFMs, (3) BFMs/F127-PTX and (4) WT; (n = 5 per treatment). At two weeks after immunization, mice were boosted with the same formulations. The mice were sacrificed at three weeks and cytokine production, intracellular cytokine staining (ICS) assay, and the expression of PD-1 and Foxp3 in CD4^+^ T cells were examined. 

#### 2.4.11. Cytokine Production Determined Using ELISA 

Single-cell suspensions were prepared from spleens of immunized mice and seeded into 96-well plates. Cells were pulsed with antigen pools for three days at 37 °C. The levels of secreted IFN-γ and IL-4 were determined using ELISA kits, according to the manufacturer’s guidelines. 

#### 2.4.12. Intracellular Cytokine Staining (ICS) Assay 

Lymphocytes harvested from immunized mice were seeded into 24-well plates and stimulated with tumor antigens for 1 h at 37 °C. Brefeldin A was then added to inhibit the secretion of cytokines into the extracellular space and the cells were co-cultured for another 4 h at 37 °C. Lymphocytes were stained with FITC-conjugated anti-mouse CD8a (FITC-CD8a) or CD4(FITC-CD4) antibodies in 50 μL PBS (containing 1% BSA) for 30 min at 4 °C. IC fixation and permeabilization buffers were added. The cells were labeled with anti-mouse PE conjugated IFN-γ or IL-4 antibodies for 30 min at 4 °C. After washing with PBS, the percentage of IFN-γ-producing CD8^+^ T cells and secreting IL-4 CD4^+^ T cells was determined using flow cytometer (BD, USA).

#### 2.4.13. Expressing PD-1 and Foxp3 of CD4^+^ T Cells

Single lymphocyte suspensions were harvested from immunized mice. Cells were seeded into 24-well plates and re-stimulated with tumor antigen for 1 h at 37 °C. Lymphocytes were stained with FITC-conjugated CD4 (FITC-CD4) and PE-CY5 labeled Foxp3 (fork-head F-box protein) or PE-labeled PD-1 anti-mouse antibodies in 50 μL PBS having 1% BSA for 30 min at 4 °C. After washing with PBS, the expression of PD-1 or Foxp3 in CD4^+^ T cells was determined using flow cytometer (BD, USA).

#### 2.4.14. Data Analysis

All data are expressed as mean ± standard deviation (SD). Statistical analysis was performed using GraphPad Prism software, and at least three independent experiments were conducted. Statistical significance was determined using *t*-test or one-way ANOVA. Statistical significance was set at *p* < 0.05.

## 3. Results

### 3.1. The Characterization of F127/PTX Micelles 

**Figure 2:**
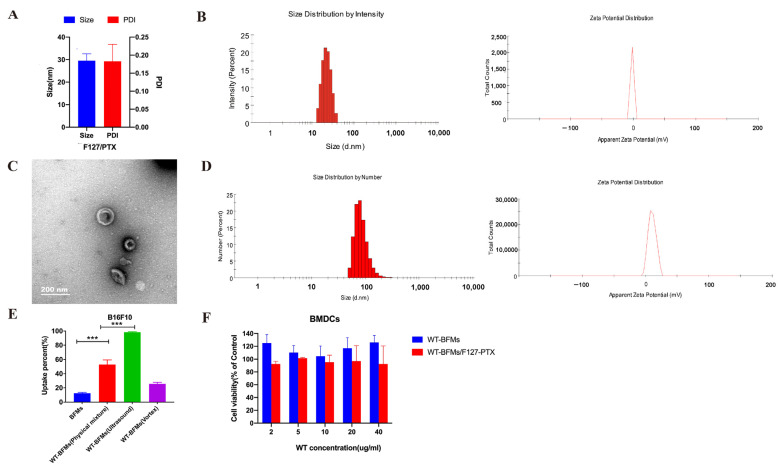
Characteristics of WT-BFMs/F127-PTX and its components. (**A**,**B**) size, PDI and zeta potential of F127/PTX. (**C**,**D**) Size, morphology and zeta potential of WT-BFMs/F127-PTX. (**E**) The uptake of WT-BFMs based on different prepare method. (**F**) An MTT assay of WT-BFMs and WT-BFMs/F127-PTX (n = 6). *** *p* < 0.001.

F127/PTX micelles were prepared using the thin-film dispersion method. The obtained F127/PTX showed a mean hydrodynamic diameter 30.1 ± 2.9 nm and a PDI of 0.185 ± 0.047 (Figure 2A). The zeta potential of F127/PTX was -1.2 mV. The encapsulation efficiency of PTX determined using HPLC was 86.53 ± 0.15%.

### 3.2. The Characterization of BFMs and Membranes Derivation Preparation 

**Figure 3:**
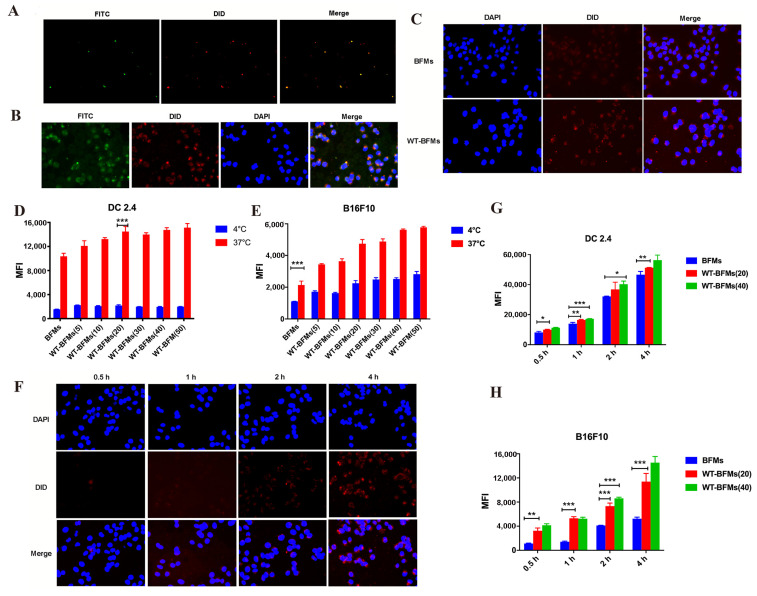
Colocalization of the FITC-WT and BFMs and uptake of BFMs and WT-BFMs in DC 2.4 cells and the cellular uptake efficiency and mechanism of WT-BFMs in DC 2.4 cells. (**A**,**B**) Colocalization of the BFMs (red channel) and the FITC-WT (green channel) observed by fluorescence microscope. (**C**) The uptake of BFMs and WT-BFMs in DC 2.4 using fluorescence microscope. BFMs were labeled with DiD (red channel) and cell nucleus were labeled with DAPI (blue channel). (**D**,**E**) The uptake of BFMs and WT-BFMs at a different temperature (4 °C and 37 °C) or at a different ratio of BFMs and WT. (**F**) The uptake of WT-BFMs in DC 2.4 using fluorescence microscope. BFMs were labeled with DiD (red channel) and cell nucleus were labeled with DAPI (blue channel).(**G**,**H**) The uptake of BFMs and WT-BFMs in DC 2.4 and B16F10 for 0.5 h, 1 h, 2 h, 4 h (of which the final concentration of WT was 20 μg/mL,40 μg/mL). Data are mean ± SD (n = 3). *** *p* < 0.001, ** *p* < 0.01, * *p* < 0.05.

The BFMs were prepared using the low-osmosis method. The mean hydrodynamic diameters, PDI and surface charge were measured. BFMs showed anionic surface charge of −39.6 ± 0.5 mv and a mean hydrodynamic diameter of 187.75 ± 23.55 nm and a PDI of 0.201 ± 0.53. WT-BFMs/F127-PTX showed a similar size (Figure 2D), whereas due to the addition of canonical WT, WT-BFMs/F127-PTX showed a higher zeta potential (11.2 ± 0.7 mV) (Figure 2D). The morphology of WT-BFMs/F127-PTX was characterized using transmission electron microscopy (Figure 2C). Colocalization of BFMs and FITC-WT was confirmed using fluorescence microscopy (Figure 3A,B). The green channel (FITC-WT) was merged with the red channel (BFMs), and the yellow color represents the form of the WT-BFMs (Figure 3A,B).

### 3.3. Cytotoxicity of Nanoparticles

MTT assay was conducted to assess the cytotoxicity of WT-BFMs and WT-BFMs/F127-PTX (n=6). These results suggested that WT-BFMs and WT-BFMs/F127-PTX were minimally toxic (Figure 2F).

### 3.4. Optimization of Composition and Uptake Efficiency of Nanoparticles

Fluorescence microscopy images demonstrated that WT-BFMs had higher uptake efficacy than BFMs in BMDCs (Figure 3C) which indicates that our strategy of WT-fusion with BFMs improves the cellular uptake of BFMS. 

The ratio of WT to BFMs was optimized based on the uptake assay. WT-BFMs were efficiently internalized by DC 2.4 and B16F10 cells (Figure 3D). The mean fluorescence intensity (MFI) of WT-BFMs was 1.6-fold higher than that of BFMs group, in which the WT concentration was 20 μg/mL in DC 2.4 cells (*p* < 0.001; Figure 3D). Additionally, the increasing MFI of WT-BFMs depended on the concentration of the WT. The uptake of WT-BFMs(50) in B16F10 cells was significantly higher than that of BFMs, which was 2.0-fold higher than the MFI of BFMs (Figure 3E).

### 3.5. Cellular Uptake Mechanism of Nanoparticles

To analyze the uptake mechanism of WT-BFMs and BFMs, and uptake assay was conducted at 4 or 37 °C. The results indicated that the internalization of WT-BFMs with different concentration of WT and BFMs was lower at 4 °C than at 37 °C (*p* < 0.001, Figure 3G,H), suggesting that the internalization of BFMs derivations occurred via an energy-dependent pathway. We also observed that the internalization of WT-BFMs depended on uptake time (Figure 3F–H).

### 3.6. Ability of Nanoparticle to Induce BMDCs Maturation and Cytokine Secretion 

**Figure 4:**
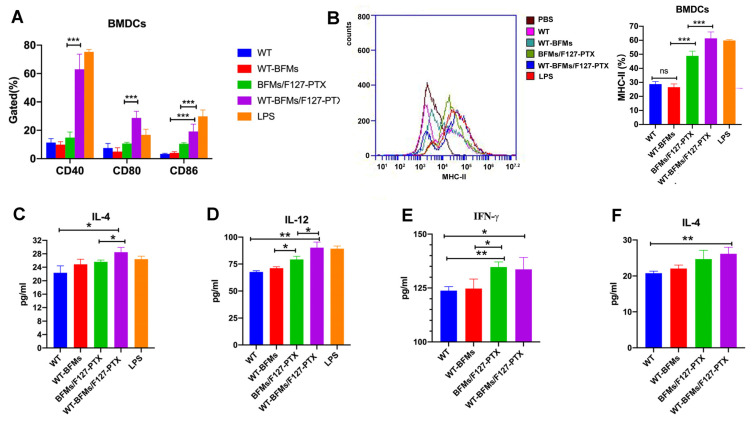
The maturation and the levels of cytokines secretion of BMDCs after pulsed with WT-BFMs/F127-PTX. (**A**) The CD40, CD80 and CD86 expression of BMDCs. (**B**) The MHC-Ⅱ expression of BMDCs. (**C**,**D**) The levels of cytokines secretion of BMDCs. (**E**,**F**) The levels of cytokines secretion of lymphocytes harvest from immunized mice which were pulsed with antigen pools for 3 days at 37 °C. Data are mean ± SD (n = 3). *** *p* < 0.001, ** *p* < 0.01, * *p* < 0.05.

The expression of costimulatory factors CD40, CD80, and CD86 on BMDCs was measured using flow cytometry (Figure 4A). Compared to cells treated with WT-BFMs (4.1%), BMDCs were stimulated to maturation status by BFMs/F127-PTX with a high expression of CD86(10.5%, *p* < 0.001, Figure 4A). BFMs/F127-PTX triggered significant BMDCs maturation. Furthermore, BMDCs activation was further improved by WT modification. The expression percentage of CD86 in the WT-BFMs/F127-PTX group (19.17%) was approximately two-folds higher than in the BFMs/F127-PTX (*p* < 0.001, Figure 4A).

MHC-Ⅱ expression in BMDCs was measured using flow cytometer (BD, East Rutherford, NJ, USA). The percentage of MHC-Ⅱ ^+^ BMDCs in the WT-BFMs group was 26.6% which was not significantly different from that of WT-BFMs. WT-BFMs/F127-PTX stimulated BMDCs express the highest MHC-Ⅱ (61.4%) and was 1.27-fold higher than ones of BFMs/F127-PTX (48.3%, *p* < 0.001, Figure 4B).

The secretions of IL-12 from BMDCs incubated with various nanoparticles was tested using ELISA. BMDCs treated with BFMs/F127-PTX showed significant increased secretion of IL-12 (Figure 4D). Additionally, the highest levels of IL-12 were observed in BMDCs co-cultured with WT-BFMs/F127-PTX (Figure 4D). Furthermore, enhanced production of IL-4 was observed in the WT-BFMs/F127-PTX group compared to the WT-BFMs group (*p* < 0.05, Figure 4C).

### 3.7. Cytokine Secretion of CD8^+^ T Cells and CD4^+^ T Cells 

The production levels of IFN-γ and IL-4 in the spleen culture supernatants of immunized mice were determined using ELISA as previously described. WT-BFMs/F127-PTX triggered up-regulation of IL-4 production (Figure 4E,F). 

### 3.8. Intracellular Cytokine Staining (ICS) Assay

**Figure 5:**
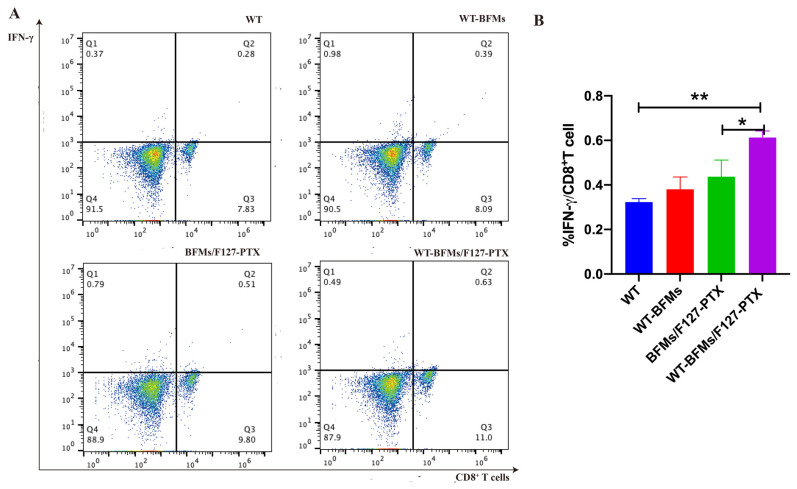
IFN-γ producing by CD8^+^ T cells. (**A**) Representative dot plots of cells stained in the assay. (**B**) Quantitation of the percentages of IFN-γ^+^/CD8^+^ T cells. Female C57BL/6 mice aged 6- to 8-weeks were administrated of the following formulations subcutaneously (1) WT-BFMs/F127-PTX; (2) WT-BFMs; (3) BFMs/F127-PTX; (4) WT; (n = 5 per treatment). At 2 weeks after immunization, mice were boosted with the same formulations. Intracellular cytokine staining (ICS) assay was conducted. The percentage of IFN-γ producing CD8^+^ T cells was determined by Flow cytometer (BD, USA), ** *p* < 0.01, * *p* < 0.05.

**Figure 6:**
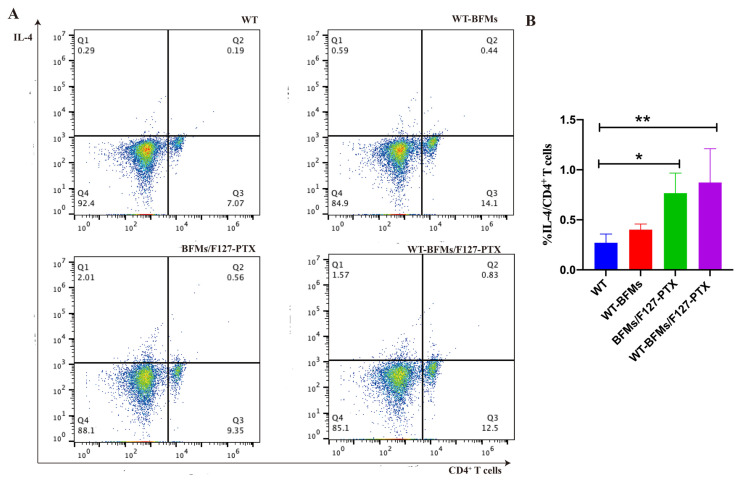
IL-4 secretion CD4^+^ T cells. (**A**) Representative dot plots of cells stained in the assay. (**B**) Quantitation of the percentages of IL-4^+^/CD4^+^ T cells. Single lymphocytes suspensions were harvested from immunized mice. Intracellular cytokine staining (ICS) assay was conducted. The percentage of IL-4 secretion CD4^+^ T cells were determined by Flow cytometer (BD, USA). ** *p* < 0.01, * *p* < 0.05.

We evaluated antigen-specific T-Cell response using intracellular staining for IFN-γ and IL-4. The frequencies of CD8^+^ T cells secreting IFN-γ and CD4^+^ T cells producing IL-4 in response to antigen in WT-BFMs/F127-PTX immunized mice (0.61% and 0.87%, respectively) were significantly higher than those in WT mice (0.30% and 0.27%, respectively) (*p* < 0.05; Figure 5A,B and Figure 6A,B). Additionally, BFMs/F127-PTX elicited IFN-γ-production by antigen-specific CD8^+^ T cells (0.44%, Figure 5A,B) and IL-4 production by antigen-specific CD4^+^ T cells (0.77%, Figure 6A,B). WT-BFMs induced higher IFN-γ -secreting CD8^+^ T cells than WT cells (0.38%, Figure 5A,B), and triggered a higher CD4^+^ Th2 response (0.40%, Figure 6A,B). IFN-γ and IL-4 production in splenocyte culture supernatant was increased after subcutaneously administration of WT-BFMs/F127-PTX. These results indicate the efficacy of WT-BFMs/F127-PTX nanoparticles in immune responses.

### 3.9. Expressing PD-1 and Foxp3 of CD4^+^ T Cells

**Figure 7:**
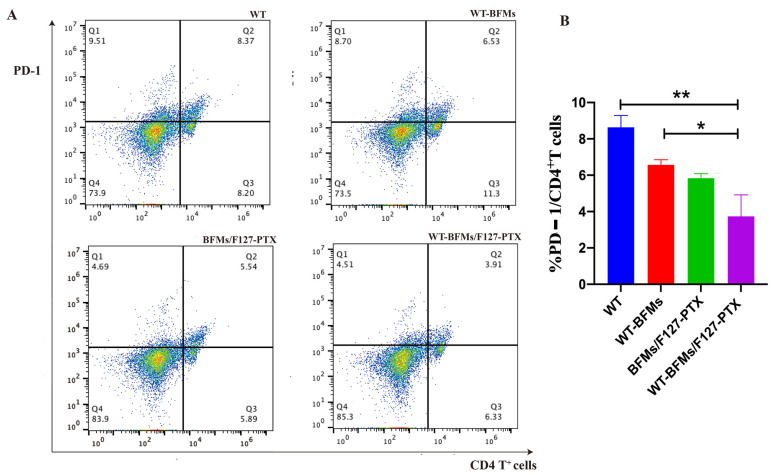
The PD-1 expression of CD4^+^ T cells. (**A**) Representative dot plots of cells stained in the assay. (**B**) Quantitation of the percentages of PD-1^+^/CD4^+^ T cells. Single lymphocytes suspensions were harvested from immunized mice and seeded into in 24-well plates and restimulated with tumor antigen for 1 h at 37 °C. Lymphocytes were stained with FITC-conjugated anti-mouse CD4 antibodies (FITC-CD4) and PE-labeled PD-1 anti-mouse antibodies in 50 μL PBS (1% BSA) for 30 min at 4 °C. After being washed with PBS, the expressing percentage of PD-1 on CD4^+^ T cells were determined by Flow cytometer. Data are mean ± SD (n = 5). ** *p* < 0.01, * *p* < 0.05.

The percentage of PD-1 expressed in CD4^+^ T cells was determined. No differences were found in the frequency of PD-1^+^/CD4^+^ T cells between WT-BFMs/F127-PTX and BFMs/F127-PTX groups (Figure 7A,B). However, the percentage of PD-1+ CD4^+^T cells was lower than that in WT and WT-BFMs (*p* < 0.01 and *p* < 0.05, Figure 7A,B).

**Figure 8:**
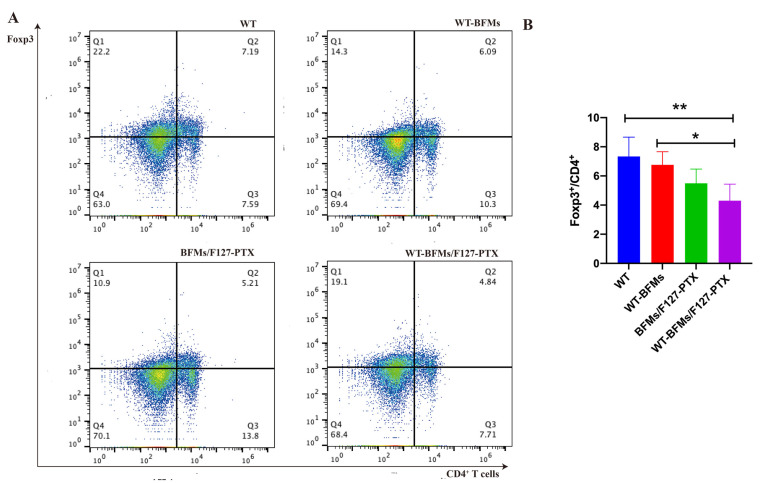
The Foxp3 expression of on CD4^+^ T cells. (**A**) Representative dot plots of cells stained in the assay. (**B**) Quantitation of the percentages of Foxp3^+^/CD4^+^.Single lymphocytes suspensions were harvested from immunized mice and seeded into in 24-well plates and restimulated with tumor antigen for 1 h at 37 °C. Data are mean ± SD (n = 5). ** *p* < 0.01, * *p* < 0.05.

Foxp3 is a marker of Tregs and regulates the development and function of T cells. The frequency of Tregs was determined by measuring CD4^+^/Foxp3 levels using a low cytometer. No differences were found in the frequency of Foxp3^+^/CD4^+^ T cells, between the WT-BFMs/F127-PTX and BFMs/F127-PTX groups (Figure 8A,B). The percentage of Foxp3^+^ CD4^+^T cells was lower than in WT and WT-BFMs (*p* < 0.01 and *p* < 0.05, Figure 8A,B).

## 4. Discussion

Melanoma caused a high number of skin cancer-related deaths globally. An effective anti-tumor vaccine is composed of tumor specific antigen, potent adjuvants and delivery carriers. Cell membranes are becoming increasingly prevalent for assisting drug delivery because of the significant benefits of avoiding host cell barriers. Additionally, biological cell membrane components make tumor cell-derived cell membranes act as antigens for vaccines [17]. However, the safety profile of tumor cell-derived membranes is usually a concern because these membranes may generate tolerance. Based on this, we deliberated on the composition of our nanoparticles. A combined treatment of antigen cell membranes with antigen peptide WT and immune-adjuvant PTX may promote the activation of antigen-specific immune responses. Therefore, we constructed vaccines by encapsulating PTX/F127 micelles with BFMs. Then, the preparation method was considered. Then, the incorporation of WT into cell membranes by ultrasound can avoid a decrease in membranes biological activity instead of synthesis. In the uptake assay, WT-BFMs/F127-PTX were formed using ultrasound (Figure 2E). Furthermore, this approach avoids the need for time-consuming and extensive research to adjust the properties of nanoparticles. Additionally, a certain concentration of WT and PTX was considered to achieve effective uptake and DC function. In our preliminary study, we only changed the concentration of WT, BFMs and F127-PTX. 

Transmission electron microscopy images showed spherical the structures of the nanoparticles (Figure 2C). The structures were likely to form a hydrated layer on the surface of the nanoparticles in the solvent; therefore, the size of WT-BFMs/F127-PTX (187.75 ± 23.55 nm) at pH 7.0 determined using DLS detector method was larger than that observed using transmission electron microscopy (approximately 160 nm). The zeta potential of BFMs in neutral media was negative, and after coating with WT, the zeta potential of WT-BFMs/F127-PTX was electropositive. The zeta potential of the resultant WT-BFMs/F127-PTX increased depending on the WT concentration.

The in vitro performance of WT-BFMs/F127-PTX on BMDCs, DC 2.4 was subsequently evaluated thoroughly to validate that WT-BFMs/F127-PTX was highly internalized by APCs. WT-BFMs could boost the uptake efficiency of BFMs owing to the addition of TAT in WT which could enhance immunocyte uptake behavior (Figure 3). 

Whether antigens and adjuvants can efficiently induce the maturation of BMDCs greatly determines vaccine immune responses. DCs can induce immune tolerance or activation depending on their subtypes [18]. CD40, CD80, and CD86 are the markers of BMDCs’ maturation. Therefore, we first evaluated the expression of the costimulatory factors CD40, CD80 and CD86 in BMDCs. Instead of acting as a cytotoxic agent to destroy cancer cells, a low dose of PTX shows low toxicity to immune cells and could induced increased expression of co-stimulatory molecules by BMDCs. Only the WT and WT-BFMs showed no appreciable BMDCs activation effect. BFMs/F127-PTX stimulated BMDCs maturation status. Notably, WT-BFMs/F127-PTX appeared to be the most effective in triggering maturation (Figure 4A). BFMs/F127-PTX also promoted the maturation of dendritic cells (Figure 4A). 

MHC-II molecules also play an important role in DCs presenting antigen peptides to naïve CD4^+^ T cells to initiate of adaptive immunity [19]. PTX mimics the action of LPS, thereby inducing MHC-II expression in DCs. The MHC-II expression in WT-BFMs/F127-PTX and LPS groups was similar (*p* > 0.05, Figure 4B) but higher than that in the other groups. Cross-presentation of the extracellular antigens to T cells with MHC-I molecules is essential for inducing strong and durable CD8^+^ T cell responses [16]. MHC-I expression on BMDCs was similar between WT-BFMs/F127-PTX and BFMs/F127-PTX (data was not shown).

The secretion of cytokines IL-12p70 and IL-4 by BMDCs is highly relevant to antitumor immunity [20]. The secretion of IL-12p70 and IL-4 was detected using ELISA kits. The results showed that the administration of WT-BFMs/F127-PTX facilitated cytokines generation (Figure 4C,D). Taken together, these data indicate that our nano-vaccine formulation WT-BFMs/F127-PTX, with PTX loading as the adjuvant, WT to provide TAT and antigen peptide, BFMs acting as vectors and tumor antigens, appears to be the most effective for stimulating the maturation of DCs.

Furthermore, intracellular staining (ICS) assays have helped to compare and estimate the ability of vaccine candidates to trigger antigen-specific immune response [11]. The Trp2 peptide and cell membrane-specific CD8^+^ and CD4^+^ T cell responses were the leading indicators for evaluating immune ability of our nanocomplexes. The proliferation of T cells of WT-BFMs/F127-PTX group is also observed in Figure 5 and Figure 6. IFN-γ produced by CD8^+^ T cells, which serves as a Th1 marker of cellular immune response, is a gold standard to evaluate CD8^+^ T cell immunity. IL-4 secreted by CD4^+^ T cells is deemed essential to elicit humoral immune response. The percentage of CD8^+^ T cells producing IFN-γ and CD4^+^ T cells secreting IL-4 was evaluated using intracellular staining. PTX enhance BMDCs functions due to signaling through the TLR-4 [15] and therefore activates the CD4^+^T cells. As expected, the nanocomplexes WT-BFMs/F127-PTX were even better at activating CD8^+^ and CD4^+^ T cells than BFMs/F127-PTX and BFMs, indicating that WT-BFMs/F127-PTX could trigger potent Th1 and Th2 immune responses.

A critical limitation of the vaccine technology is immune suppression. Insidious suppression of the immune system may be responsible for the higher mortality associated with melanoma. Melanoma cells impair immune cell trafficking and up-regulate immune suppressor cells or cytokines, such us regulatory T cells (Tregs) and myeloid derived suppressor cells (MDSCs). Tregs maintain immune homeostasis and tolerance to self [21]. Tregs can arise under pathological conditions and decrease tumor-specific T-effector (Teff) cells and NK cell responses [22]. Foxp3 is necessary for Tregs’ suppressive functions. Other molecules central to tolerance are the programmed cell death protein 1 (PD-1) and its ligand PD-L1 which could lead to T cells exhaustion and immunosuppression milieu [23]. Fanelli et al. reported that highly suppressive T cells were induced by PD-1 ligation on human CD25-depleted CD4^+^ T cells [21]. Whether BFMs cause immunosuppression remains unclear. We subsequently explored the expression of PD-1 or Foxp3 in CD4^+^ T cells. Both PD-1 and Foxp3 expression on CD4^+^ T cells of WT-BFMs/F127-PTX decreased (Figure 7 and Figure 8).

In summary, WT-BFMs/F127-PTX induced potent antigen-specific humoral and cellular immune responses. Therefore, our research presents an innovative way to fabricate cell membranes and we expect our coating strategies to be effective in protecting against other diseases.

## 5. Conclusions

In this study, we prepared WT modified BFMs-Camouflaged micelles. The formed biomimetic nanoparticles WT-BFMs/F127-PTX showed outstanding performance in uptake and inducing the maturation of BMDCs. It was evident that WT-BFMs/F127-PTX delivered subcutaneously elicited robust antigen-specific cellular and humoral immune responses at the same time resulting higher level of secreting cytokine IFN-γ and IL-4 by T cells and decreased the PD-1 or Foxp3 expression on CD4^+^ T cells. Thus, WT-BFMs/F127-PTX is a promising vaccine candidate to improve immunotherapy for melanoma.

## Data Availability

Not applicable.
